# Unlocking the Potential: Investigating Dental Practitioners’ Willingness to Embrace Artificial Intelligence in Dental Practice

**DOI:** 10.7759/cureus.55107

**Published:** 2024-02-27

**Authors:** Parameswari Royapuram Parthasarathy, Santosh R Patil, Ali Azhar Dawasaz, Fawaz Abdul Hamid Baig, Mohmed Isaqali Karobari

**Affiliations:** 1 Centre for Global Health Research, Saveetha Medical College and Hospitals, Saveetha Institute of Medical and Technical Sciences, Chennai, IND; 2 Department of Oral Medicine and Radiology, Chhattisgarh Dental College and Research Institute, Rajnandgaon, IND; 3 Department of Diagnostic Dental Sciences, College of Dentistry, King Khalid University, Abha, SAU; 4 Department of Oral and Maxillofacial Surgery, College of Dentistry, King Khalid University, Abha, SAU; 5 Dental Research Unit, Centre for Global Health Research, Saveetha Institute of Medical and Technical Sciences, Chennai, IND

**Keywords:** perceptions, attitudes, adoption, dental professionals, dentistry, artificial intelligence

## Abstract

Background: Artificial intelligence (AI) holds significant promise for transforming healthcare delivery, including dentistry. However, the successful integration of AI into dental practice necessitates an understanding of dental professionals' perspectives, attitudes, and readiness to adopt AI technology. This study aimed to explore dental professionals' perceptions, attitudes, and practices regarding AI adoption in dentistry.

Methods: This cross-sectional study was conducted among 256 dental professionals using an online questionnaire. Participants were assessed for familiarity with AI technology, perceived barriers to adoption, attitudes towards AI, current usage patterns, and factors influencing adoption decisions. Data are analysed using descriptive statistics, including frequencies, percentages, means, and standard deviations. Inferential statistics, such as chi-square tests and regression analysis, were employed to examine associations between variables and identify predictors of AI adoption in dentistry.

Results: The study surveyed 256 dental professionals from various regions across India, primarily aged 30 to 50 years (mean age: 42.6), with a nearly equal gender split (male: 48.4%, female: 51.6%) and high educational attainment (67.8% with master's or doctoral degrees). Private practices were predominant (56.3%). The diagnostic algorithms and treatment planning software were well known (77.3% and 70.3% familiarity, respectively). Technical concerns (average score: 3.82 ± 0.68) were the main barriers to AI adoption, followed by financial considerations (average score: 3.45 ± 0.72), ethical and legal issues (average score: 3.21 ± 0.65), and organizational factors (average score: 3.67 ± 0.71). Despite these concerns, most participants had positive attitudes towards AI (70.3% agreed). Current usage varied, with diagnostic support and administrative tasks being the most common (44.5% and 82.8% usage, respectively). Perceived utility (average score: 4.12 ± 0.75) and ease of use (average score: 3.98 ± 0.69) significantly influenced adoption, as identified by regression analysis (perceived utility: β = 0.342, p < 0.001; ease of use: β = 0.267, p = 0.005).

Conclusion: This study provides valuable insights into AI adoption in dentistry, highlighting the multifaceted nature of barriers and facilitators that influence dental professionals’ adoption decisions. Strategies to promote AI adoption should address practical considerations, ethical concerns, and educational needs to facilitate the integration of AI technology into dental practices.

## Introduction

Artificial intelligence (AI) is revolutionizing healthcare by offering innovative solutions to enhance patient care, optimize clinical decision-making, and streamline administrative processes [1,2]. In dentistry, the potential of AI to transform various aspects of dental practice, from diagnosis and treatment planning to patient management, presents exciting opportunities for improving oral healthcare delivery [3,4]. However, the successful integration of AI into dental practice requires a comprehensive understanding of dental professionals' perspectives, attitudes, and readiness to adopt AI technology [5].

Although there has been an increasing interest in incorporating AI into healthcare, research examining the adoption of AI in dentistry is scarce. It is essential to understand the viewpoints of dental professionals to recognize potential obstacles and develop AI solutions that cater to the distinct requirements and procedures of dental practice. Hence, this study aimed to fill this gap by investigating the perceptions, attitudes, and practices of dental professionals regarding the integration of AI in dentistry.

The integration of AI technology into dentistry has the potential to revolutionize patient care, enhance diagnostic accuracy, and improve treatment outcomes [6]. By leveraging AI algorithms for image analysis, predictive modelling, and decision support, dental professionals can augment their clinical capabilities and provide personalized and efficient care to patients [7]. Furthermore, AI-enabled administrative tools can streamline practice management tasks, optimize resource allocation, and improve overall practice efficiency [8].

Understanding the various factors that impact AI adoption among dental professionals is crucial for leveraging the advantages of AI technology in the field of dentistry and overcoming obstacles to its implementation.

The aims of this study were twofold: first, to evaluate dental professionals' familiarity with different AI applications in dentistry, such as diagnostic algorithms, treatment planning software, robotic assistance, and administrative tools; and second, to explore the perceived barriers, attitudes, and existing usage patterns that affect AI adoption decisions among dental professionals. By shedding light on the aspects that drive or hinder AI adoption in dentistry, this study aimed to develop effective strategies for promoting the appropriate and successful incorporation of AI technology into dental practice.

## Materials and methods

The total sample size calculation was carried out by applying a sample size calculator (https://selectstatistics.co.uk/calculators/sample-size-calculatorpopulation-proportion/). The error margin was kept at 5%, the confidence interval (CI) was 90%, and the sample proportional expected was set at 75%. The sample size obtained was 203; the current study included a total of 256 participants.

Participants selection

The criteria for selection were contrived to encompass a broad range of dental professionals while simultaneously ensuring an adequate sample size for rigorous analysis. The inclusion criteria comprised licensed dentists, dental hygienists, and dental assistants who were actively engaged in dental practice. The aim was to include participants from various geographical regions, practice settings (e.g., private clinics, academic institutions, public health clinics), and years of experience to obtain comprehensive insights.

Ethical considerations

Before initiating data collection, all participants received comprehensive information about the study's objectives, procedures, and potential risks and benefits. Informed consent was obtained from each participant before their participation in the study. To maintain the confidentiality of participants' responses, all data were anonymized, and measures were taken to protect their privacy. Ethical approval was granted by the Institute Review Board, Saveetha Medical College and Hospitals, SIMATS, Chennai, India, with an ethical approval code (086/01/2024/Faculty/SRB/SMCH).

Data collection instruments

The development of the questionnaire was a thorough process that commenced with a comprehensive examination of existing literature on AI adoption in dentistry and related areas. Drawing on validated instruments from prior studies and consulting with experts, a detailed questionnaire was designed to capture the intricacies of dental professionals' perceptions and practices regarding AI technology.

Domains covered

Demographics

Age, gender, educational background, years of practice, practice setting, and specialty areas were included to provide a comprehensive profile of the participants.

Familiarity With AI Technology

Questions were designed to assess participants' level of familiarity with various AI applications in dentistry, including diagnostic algorithms, treatment planning software, robotic assistance, and administrative tools.

Perceived Barriers to Adoption

Participants identified and rated the significance of potential barriers hindering the adoption of AI technology in their dental practice. These barriers encompassed technical concerns (e.g., reliability, accuracy), financial considerations (e.g., cost, return on investment), ethical and legal issues (e.g., data privacy, liability), as well as organizational and cultural factors (e.g., resistance to change, lack of training).

Attitudes Towards AI in Dentistry

Attitudinal items aimed to gauge participants' overall perceptions of AI technology in dentistry, including perceived benefits, concerns, and willingness to adopt AI-enabled solutions in their practice.

Current Usage Patterns

Participants were asked about their current utilization of AI technology in various aspects of dental practice, such as diagnostic support, treatment planning, patient management, and administrative tasks. This section also explored the frequency and extent of AI integration in their day-to-day practice.

Factors Influencing Adoption Decisions

This domain delves into the factors influencing participants' decisions regarding the adoption or rejection of AI technology. Factors examined included perceived utility, ease of use, compatibility with existing workflows, peer influence, training and support availability, and patient acceptance.

Pre-Testing and Validation

Before its full-scale implementation, the questionnaire was subjected to extensive pre-testing among a group of dental professionals. This process aimed to assess the clarity, comprehensibility, and relevance of each questionnaire item. Feedback from participants was solicited to identify any ambiguities or areas that required clarification. Based on the pre-test results, necessary revisions were made to optimize the questionnaire for subsequent data collection efforts.

Data collection procedures

Participants were given the option to complete the survey using Google Forms, a secure online platform. Each participant received a unique link to access the survey, ensuring anonymity. The survey consisted of structured questions covering demographics, familiarity with AI, perceived barriers, and adoption factors. Reminder emails were sent to enhance response rates. Instructions were provided within the form to guide participants through the survey process. Data integrity was ensured through checks for completeness and consistency.

Data analysis

Data analysis was performed using the statistical software package SPSS, Version 22.0 (IBM Corp., Armonk, NY). The data obtained from the questionnaires were analyzed using descriptive statistics, including frequencies, percentages, means, and standard deviations. Inferential statistics, such as chi-square tests and regression analysis, were employed to examine associations between variables and identify predictors of AI adoption in dentistry.

## Results

Demographics

The study recruited a diverse sample of 256 dental professionals, reflecting a range of ages, genders, educational backgrounds, and practice settings (Table 1). The surveyed dental professionals were from various regions across India, representing a nationwide sample. This pan-India approach aimed to capture a diverse range of perspectives and experiences from dental professionals practicing in different parts of the country.

**Table 1:** Participant demographics

Demographic characteristic	Frequency (n=256)	Percentage
Age (years)		
<30	45	17.6%
30–40	72	28.1%
41–50	58	22.7%
51–60	55	21.5%
>60	26	10.2%
Gender		
Male	132	51.6%
Female	124	48.4%
Educational background		
Bachelor's degree	68	26.6%
Master's degree	102	39.8%
Doctoral degree	86	33.6%
Practice setting		
Private Practice	138	53.9%
Academic Institution	62	24.2%
Public Health Clinic	56	21.9%

The majority of participants fell within the age range of 30-50 years, with a nearly equal distribution between male and female participants. Educational attainment was high, with a significant proportion holding a master's or doctoral degree. Private practice emerged as the dominant practice setting, with over half of the participants indicating it as their primary practice environment. The demographic profile of the participants suggested a broad representation of dental professionals, providing a comprehensive basis for understanding their perceptions and practices regarding AI adoption in dentistry.

Familiarity with AI technology

In terms of familiarity with AI technology, the participants demonstrated varying levels of acquaintance across different AI applications (Table 2). Diagnostic algorithms and treatment planning software were relatively well-known among the participants, with approximately 77.3% and 70.3% reporting familiarity, respectively. However, robotic assistance and administrative tools were less familiar, with 56.6% and 82.8% of the participants indicating familiarity, respectively. These findings highlight differences in awareness of and exposure to AI technology within the dental professional community, suggesting potential variations in readiness for the adoption and integration of AI into dental practice.

**Table 2:** Familiarity with artificial intelligence technology

AI application	Familiar (n=256)	Unfamiliar (n=256)
Diagnostic algorithms	198 (77.3%)	58 (22.7%)
Treatment planning software	180 (70.3%)	76 (29.7%)
Robotic assistance	145 (56.6%)	111 (43.4%)
Administrative tools	212 (82.8%)	44 (17.2%)

Perceived barriers to AI adoption

Perceived barriers to AI adoption were assessed, with participants rating technical concerns as the most significant barrier, followed by financial considerations (Table 3). On a scale of 1 to 5, where higher scores indicate greater significance, technical concerns received an average score of 3.82 ± 0.68, while financial considerations received a score of 3.45 ± 0.72. Ethical and legal issues were also noted as relevant barriers, albeit to a lesser extent, with an average score of 3.21 ± 0.65. Organizational and cultural factors were rated as moderately significant barriers, with an average score of 3.67 ± 0.71. These findings underscore the multifaceted nature of barriers hindering the adoption of AI in dentistry, encompassing the technical, financial, ethical, and organizational dimensions.

**Table 3:** Perceived barriers to adoption

Barrier	Significance (mean ± SD)
Technical concerns	3.82 ± 0.68
Financial considerations	3.45 ± 0.72
Ethical and legal issues	3.21 ± 0.65
Organizational and cultural factors	3.67 ± 0.71

Attitudes towards AI in dentistry

Participants generally expressed positive attitudes towards AI in dentistry, with the majority agreeing that AI has potential benefits for improving dental practice (Table 4). However, concerns about the ethical implications of AI in dentistry were notable, with 50.0% of the participants expressing concern. Despite these concerns, a significant proportion of the participants indicated a willingness to adopt AI-enabled solutions in their dental practice. For example, 70.3% agreed that they were willing to adopt AI technology. These findings suggest a nuanced perspective among dental professionals that acknowledges the potential benefits and ethical considerations associated with AI adoption.

**Table 4:** Attitudes towards artificial intelligence in dentistry

Attitude	Agree (n=256)	Neutral (n=256)	Disagree (n=256)
AI has potential benefits for improving dental practice.	212 (82.8%)	36 (14.1%)	8 (3.1%)
I am concerned about the ethical implications of AI in dentistry.	128 (50.0%)	92 (36.0%)	36 (14.1%)
I am willing to adopt AI-enabled solutions in my dental practice.	180 (70.3%)	56 (21.9%)	20 (7.8%)

Current usage patterns

The current usage patterns of AI technology in dental practice revealed varying levels of integration across different applications (Table 5). While a substantial number of participants reported using AI for diagnostic support and administrative tasks, fewer utilized AI for treatment planning and patient management. For instance, 44.5% of the participants reported using AI for diagnostic support, while only 25.8% reported using AI for patient management. These findings highlight variations in the adoption and utilization of AI technology within different aspects of dental practice, indicating opportunities for further exploration and intervention to promote broader integration.

**Table 5:** Current usage patterns

AI application	Currently using (n=256)	Not currently using (n=256)
Diagnostic support	114 (44.5%)	142 (55.5%)
Treatment planning	98 (38.3%)	158 (61.7%)
Patient management	66 (25.8%)	190 (74.2%)
Administrative tasks	152 (59.4%)	104 (40.6%)

Factors influencing adoption decisions

The factors influencing adoption decisions were examined, with perceived utility and ease of use emerging as the most influential factors (Table 6). Participants considered compatibility with existing workflows, availability of training and support, peer influence, and patient acceptance to be important considerations in their decision-making process. Perceived utility received the highest average score of 4.12 ± 0.75, followed by ease of use with a score of 3.98 ± 0.69. These findings underscore the importance of addressing practical considerations and user experience in promoting the adoption of AI technology in dentistry.

**Table 6:** Factors influencing adoption decisions

Factor	Influence level (mean ± SD)
Perceived utility	4.12 ± 0.75
Ease of use	3.98 ± 0.69
Compatibility with workflows	3.86 ± 0.72
Peer influence	3.45 ± 0.68
Training and support	3.78 ± 0.71
Patient acceptance	3.64 ± 0.66

Associations between gender and attitudes towards AI

Chi-square tests conducted to examine the associations between gender and attitudes towards AI among dental professionals revealed no significant relationship between gender and perceptions of AI benefits in dentistry (χ² = 0.129, p = 0.719) or concerns about AI ethics (χ² = 0.157, p = 0.692). However, a borderline significant association was found between gender and willingness to adopt AI-enabled solutions in dental practice (χ² = 3.273, p = 0.195), suggesting a slight trend where males were more inclined towards adoption than females (Table 7).

**Table 7:** Associations between gender and attitudes towards artificial intelligence

Attitude	Male (n=132)	Female (n=124)	Total (n=256)	Chi-square	p-value
AI has potential benefits for improving dental practice.					
Agree	108 (81.8%)	104 (83.9%)	212 (82.8%)	0.129	0.719
Neutral	16 (12.1%)	20 (16.1%)	36 (14.1%)		
Disagree	8 (6.1%)	0 (0.0%)	8 (3.1%)		
I am concerned about the ethical implications of AI in dentistry.					
Agree	64 (48.5%)	64 (51.6%)	128 (50.0%)	0.157	0.692
Neutral	46 (34.8%)	46 (37.1%)	92 (36.0%)		
Disagree	22 (16.7%)	14 (11.3%)	36 (14.1%)		
I am willing to adopt AI-enabled solutions in my dental practice.					
Agree	100 (75.8%)	80 (64.5%)	180 (70.3%)	3.273	0.195
Neutral	24 (18.2%)	32 (25.8%)	56 (21.9%)		
Disagree	8 (6.1%)	12 (9.7%)	20 (7.8%)		

Factors influencing adoption decisions

Regression analysis identified significant factors influencing adoption decisions, notably "perceived utility" (β = 0.342, p < 0.001) and "ease of use" (β = 0.267, p = 0.005), indicating that participants who perceived AI as beneficial and easy to use were more likely to adopt. "Compatibility with workflows" showed a positive relationship with adoption decisions (β = 0.198, p = 0.058), although it was not statistically significant at the conventional level (Table 8).

**Table 8:** Factors influencing adoption decisions

Factor	Beta coefficient	Standard error	t-value	p-value
Perceived utility	0.342	0.078	4.385	<0.001
Ease of use	0.267	0.094	2.845	0.005
Compatibility with workflows	0.198	0.104	1.904	0.058
Peer influence	0.124	0.087	1.429	0.155
Training and support	0.176	0.092	1.913	0.057
Patient acceptance	0.091	0.102	0.893	0.373

## Discussion

This study provides valuable insights into the perceptions, attitudes, and practices of dental professionals regarding the adoption of AI technology in dentistry. The findings shed light on various aspects of AI adoption, including familiarity with AI applications, perceived barriers, current usage patterns, and the factors influencing adoption decisions.

This study revealed varying levels of familiarity among dental professionals with different AI applications in dentistry. Diagnostic algorithms and treatment planning software are relatively well known, whereas robotic assistance and administrative tools are less familiar. These findings are consistent with previous studies highlighting the diverse landscape of AI familiarity among healthcare professionals [9,10]. They underscored the need for targeted education and training programmes to enhance dental professionals' understanding and utilization of AI technology in various aspects of dental practice.

Technical concerns emerged as the most significant barrier to AI adoption, followed by financial considerations, ethical and legal issues, and organizational and cultural factors. These findings align with prior research indicating that technical challenges such as the reliability and accuracy of AI algorithms are primary concerns among healthcare professionals [11]. Financial constraints and ethical considerations, including data privacy and liability, also pose substantial barriers to AI adoption in dentistry [7,12]. Addressing these barriers requires interdisciplinary collaboration, regulatory frameworks, and investment in AI infrastructure to ensure the safe and effective integration of AI technology into dental practice.

Despite concerns, dental professionals generally expressed positive attitudes towards AI in dentistry, acknowledging its potential benefits in improving patient care and practice efficiency. This finding resonates with other healthcare domains, indicating a growing recognition of AI as a valuable tool for augmenting clinical decision-making and enhancing patient outcomes [13,14]. However, ethical considerations surrounding AI, such as accountability, transparency, and bias, remain critical concerns that warrant careful consideration and ethical oversight in AI development and deployment [12].

The use of AI in dentistry offers significant potential for improving diagnostic accuracy, treatment planning, and patient care. However, the incorporation of AI in dentistry is prone to various biases, including algorithmic, selection, confirmation, data, and ethical biases. Algorithmic bias can occur when training data are skewed, leading to inaccuracies or disparities in the AI-generated recommendations. Selection bias may arise when training data do not represent the diversity of patient populations or healthcare settings, thereby affecting the generalizability of AI models. Confirmation bias can impact AI-driven diagnostic tools by reinforcing existing beliefs or preferences, potentially resulting in missed diagnoses or inappropriate treatment. Data bias can perpetuate disparities in dental health outcomes due to the unequal representation of certain groups or characteristics in the training data. Ethical bias can shape AI-driven decision-making processes based on subjective interpretations of ethical principles or societal values. To address bias in AI-driven dentistry, a comprehensive approach that encompasses transparent algorithm development, careful curation of training data, ongoing evaluation of model performance, and active engagement with stakeholders to promote fairness, transparency, and inclusivity in dental care delivery is needed. By mitigating biases in AI systems, dental professionals can harness the potential of technology to enhance clinical practice and optimize patient outcomes.

This study revealed variations in the adoption and utilization of AI technology in different aspects of dental practice. While a substantial number of participants reported using AI for diagnostic support and administrative tasks, fewer utilized it for treatment planning and patient management. These findings suggest that AI adoption in dentistry is still in its nascent stages, with opportunities for further exploration and integration into clinical workflows [6]. Strategies to promote broader adoption may include incentivizing AI implementation, providing training and support, and fostering collaboration between dental professionals and AI developers to co-create AI-enabled solutions tailored to dental practice needs [3,7].

Perceived utility and ease of use have emerged as the most influential factors driving adoption decisions among dental professionals. Compatibility with existing workflows, availability of training and support, peer influence, and patient acceptance also play significant roles in shaping adoption decisions. These findings underscore the importance of addressing practical considerations and user experience in facilitating the successful adoption of AI technology in dentistry [15]. Furthermore, fostering a culture of innovation and continuous learning within dental practices can promote a positive attitude towards AI adoption and facilitate the integration of AI into routine clinical practice [16].

The study found no significant relationship between sex and perceptions of AI benefits or concerns about AI ethics among dental professionals. However, a borderline significant association was found between gender and willingness to adopt AI-enabled solutions in dental practice, with males showing a slight trend towards a higher adoption propensity. While gender differences in technology adoption have not been well documented, further research is needed to explore the underlying factors driving these differences and to develop targeted interventions to promote gender-inclusive AI adoption strategies in dentistry.

Implications for practice and policy

The findings of this study have several implications for dental practice and policy. First, there is a need for educational initiatives to enhance dental professionals' knowledge and skills regarding AI technology. Continuing education programmes, workshops, and online resources can help bridge the gap between AI theory and practice and empower dental professionals to leverage AI to improve patient care [17]. Second, regulatory frameworks and guidelines should be established to address the ethical and legal considerations surrounding AI use in dentistry, including data privacy, security, and liability. Collaborative efforts between policymakers, regulatory bodies, industry stakeholders, and dental professionals are essential to ensuring the responsible and ethical deployment of AI in dental practice [18].

Limitations and future directions

Although this study provides valuable insights into AI adoption in dentistry, several limitations should be acknowledged. First, the study's cross-sectional design limits the causal inference and generalizability of the findings. Longitudinal studies should track changes in attitudes and adoption patterns over time and assess the long-term impact of AI integration on dental practice and patient outcomes. Second, self-report measures may be subject to social desirability bias and may not accurately reflect actual AI usage or behavior. Future research should employ objective measures such as usage logs or observational studies to validate self-reported data and provide a more comprehensive understanding of AI adoption in dentistry.

## Conclusions

This study contributes to our understanding of AI adoption in dentistry by examining dental professionals' perceptions, attitudes, and practices regarding AI technology. The findings underscore the multifaceted nature of AI adoption, highlighting the technical, financial, ethical, and organizational considerations that influence adoption decisions. By addressing these barriers and promoting a culture of innovation and collaboration, dental professionals can harness the potential of AI to enhance patient care and advance dentistry in the digital age.
